# Supplementary figures and images for: Maternal SMCHD1 controls both imprinted Xist expression and imprinted X chromosome inactivation
Source: Epigenetics Chromatin. 2022 Jul 18;15:26. doi: 10.1186/s13072-022-00458-3 (PMC9290310; doi:10.1186/s13072-022-00458-3)

## males

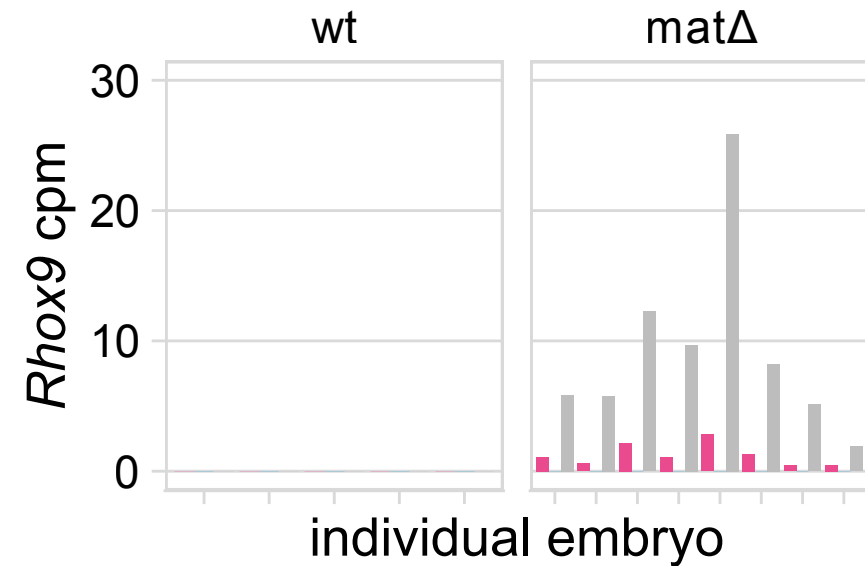

## females

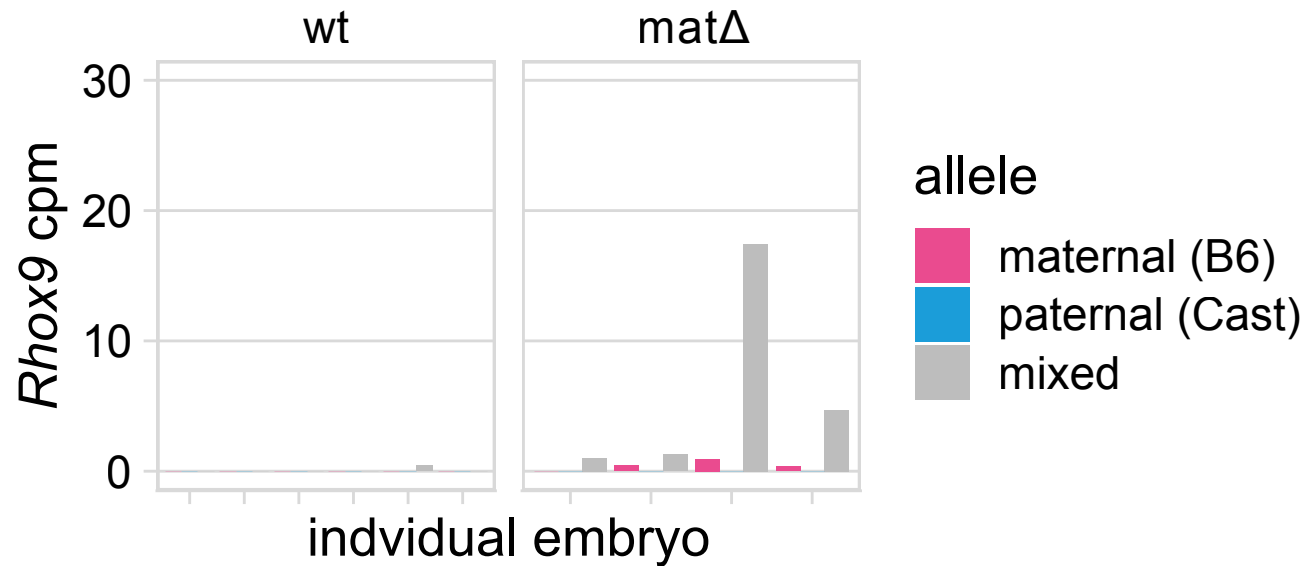

Supplement: Supplementary file 3 — Additional file 3. Supplementary Figure 1: Rhox9 expression in individual male and female wt and Smchd1matΔ E2.75 embryos. cpm: counts per million (of total library size before haplotyping). “Mixed” counts refer to counts without haplotyping. Females: n = 6 wt and 4 matΔ; males: n = 5 wt and 8 matΔ. [file 13072_2022_458_MOESM3_ESM.pdf]

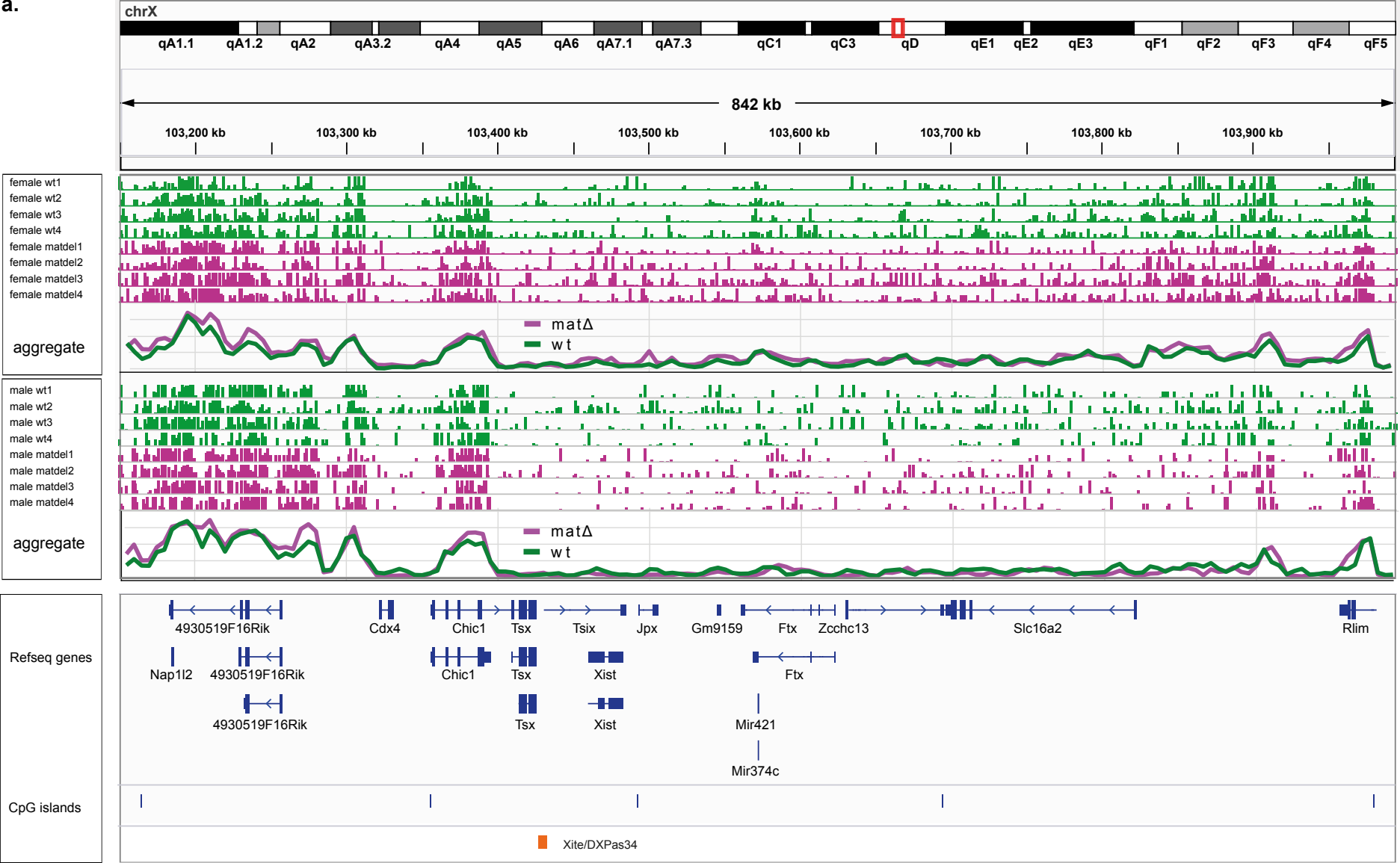

Supplement: Supplementary file 4 — Additional file 4. Supplementary Figure 2: DNA methylation at the X inactivation center in female and male E2.75 wt embryos and Smchd1matΔ embryos. Histogram tracks show methylation in single embryos at individual CpG sites (0–100%, 4 wild-type and 4 matΔembryos shown for each sex). Note that coverage in single-embryo whole-genome bisulfite sequencing is sparse, only 0–2X. The aggregate line plots show the average methylation per genotype across 10 kb windows (sliding by 5 kb, 0–100%). Females: n=6 wt and 4 matΔ; males: n=5 wt and 8 matΔ. [file 13072_2022_458_MOESM4_ESM.pdf]

female

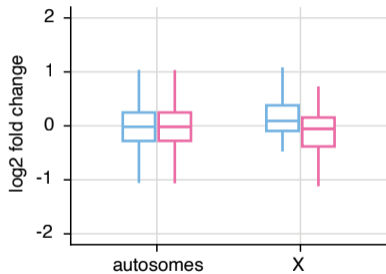

male

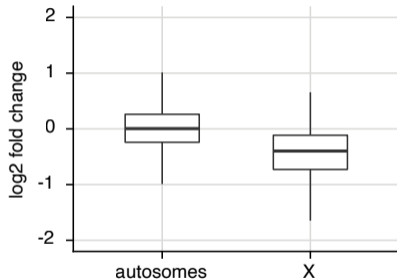

Maternal (b6) allele  
Paternal (cast) allele

Supplement: Supplementary file 5 — Additional file 5. Supplementary Figure 3: distribution of Smchd1matΔ vs wt gene expression log2 fold changes on autosomes and the X chromosome for male and female E2.75 embryos, retaining only matΔembryos with loss of Xist imprinting. [file 13072_2022_458_MOESM5_ESM.pdf]

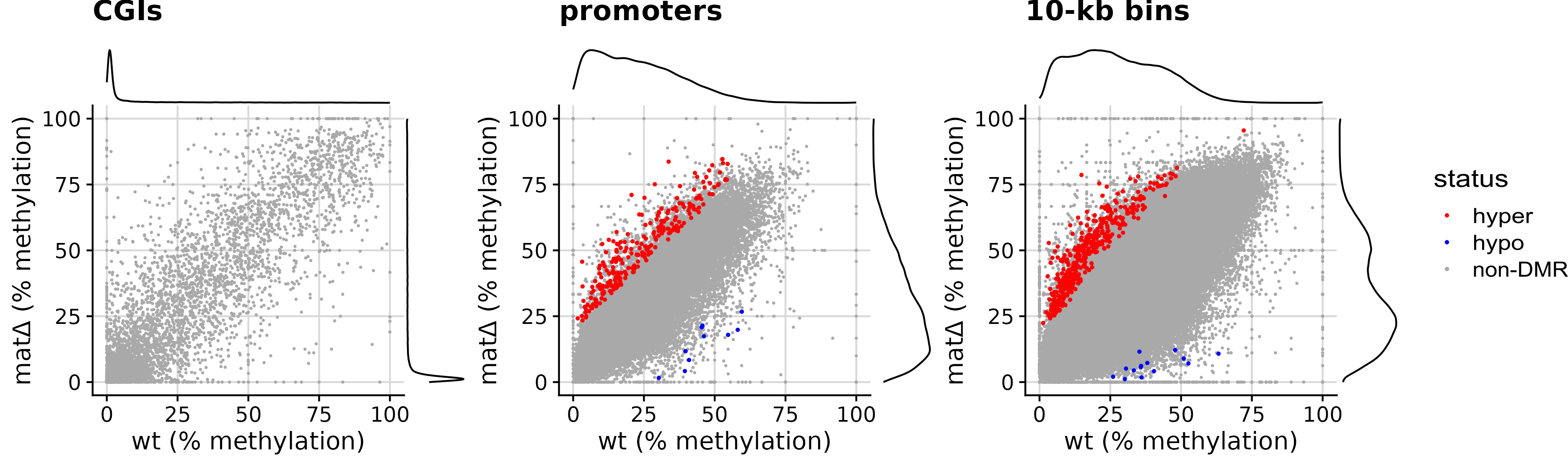

Supplement: Supplementary file 6 — Additional file 6. Supplementary Figure 4: Whole-genome differential methylation analysis between female Smchd1matΔ and wild-type E2.75 embryos. For CpG islands (CGIs, 13k regions), promoters (-4 kb to +1 kb regions, 52k regions) and 10-kb windows (sliding by 5 kb, 500k regions), the average methylation level in wild types is plotted against the average methylation in Smchd1matΔ embryos. Significant Differentially Methylated Regions (DMRs, FDR 20%) are coloured in red (hypermethylation) or blue (hypomethylation). Females: n = 6 wt and 4 matΔ. [file 13072_2022_458_MOESM6_ESM.png]
